# Prolonged Hospital Stay in Hypertensive Patients: Retrospective Analysis of Risk Factors and Interactions

**DOI:** 10.3390/nursrep15030110

**Published:** 2025-03-19

**Authors:** Stanisław Surma, Michał Czapla, Izabella Uchmanowicz, Raúl Juárez-Vela, Łukasz Pietrzykowski, Bartosz Uchmanowicz, Marcin Leśkiewicz, Krzysztof Griesmann, Michał Burzyński, Jacek Smereka, Łukasz Lewandowski

**Affiliations:** 1 Department of Internal Medicine and Clinical Pharmacology, Medical University of Silesia, 40-752 Katowice, Poland; stanislaw.surma@ptlipid.pl; 2 Department of Emergency Medical Service, Faculty of Nursing and Midwifery, Wroclaw Medical University, 51-618 Wroclaw, Poland; krzysztof.griesmann@umw.edu.pl (K.G.); michal.burzynski@umw.edu.pl (M.B.); jacek.smereka@umw.edu.pl (J.S.); 3 Group of Research in Care (GRUPAC), Faculty of Health Sciences, University of La Rioja, 26006 Logroño, Spain; raul.juarez@unirioja.es; 4 Institute of Heart Diseases, University Hospital, 50-566 Wroclaw, Poland; 5 Department of Nursing, Faculty of Nursing and Midwifery, Wroclaw Medical University, 51-618 Wroclaw, Poland; izabella.uchmanowicz@umw.edu.pl (I.U.); bartosz.uchmanowicz@umw.edu.pl (B.U.); 6 Centre for Cardiovascular Health, Edinburgh Napier University, Sighthill Campus, Edinburgh EH11 4DN, UK; 7 Department of Cardiac Rehabilitation and Health Promotion, Nicolaus Copernicus University in Torun, Collegium Medicum in Bydgoszcz, 85-094 Bydgoszcz, Poland; lukasz.pietrzykowski@cm.umk.pl; 8 Department of Emergency Medicine, Wroclaw Medical University, 50-556 Wroclaw, Poland; marcin.leskiewicz@umw.edu.pl; 9 Department of Medical Biochemistry, Wroclaw Medical University, 50-368 Wroclaw, Poland; lukasz.lewandowski@umw.edu.pl

**Keywords:** hypertension, length of hospital stay, cardiovascular risk factors

## Abstract

**Background/Objectives**: Arterial hypertension (HT) is a leading modifiable risk factor for cardiovascular diseases, often contributing to prolonged lengths of hospital stay (LOHS), which place significant strain on healthcare systems. This study aimed to analyze the factors associated with prolonged lengths of hospital stay in patients with HT, focusing on key biochemical and clinical predictors. **Methods**: This retrospective study included 356 adult patients hospitalized in the Cardiology Department of the University Hospital in Wroclaw, Poland, between January 2017 and June 2021. Data collected included demographic characteristics, body mass index (BMI), comorbidities, and laboratory parameters. Logistic regression models were used to identify predictors of prolonged LOHS, defined as four or more days, and to evaluate interactions between variables. **Results**: Lower levels of low-density lipoprotein cholesterol (LDL-c) and elevated concentrations of high-sensitivity C-reactive protein (hsCRP) were identified as significant predictors of prolonged LOHS, with each 1 mg/dL decrease in LDL-c increasing the odds of prolonged LOHS by 1.21% (*p* < 0.001) and each 1 mg/L increase in hsCRP raising the odds by 3.80% (*p* = 0.004). An interaction between sex and heart failure (HF) was also observed. Female patients with HF had 3.995-fold higher odds of prolonged LOHS compared to females without HF (*p* < 0.001), while no significant difference was found among male patients with or without HF (*p* = 0.890). **Conclusions**: The predictors of prolonged LOHS in patients with HT include lower levels of LDL-c, elevated hsCRP, and the interaction between sex and heart failure (HF). Specifically, female patients with HF demonstrated significantly higher odds of prolonged LOHS compared to females without HF, while this relationship was not observed in male patients.

## 1. Introduction

Arterial hypertension (HT) remains one of the most significant modifiable risk factors for cardiovascular diseases (CVDs), which continue to top global lists of morbidity and mortality causes [1,2]. Despite ongoing progress in diagnosis and therapeutic management, recent epidemiological reports suggest that the burden of hypertension remains alarmingly high, affecting a growing number of adults worldwide [3,4]. Poorly controlled hypertension predisposes individuals to a range of serious complications—including myocardial infarction, heart failure, chronic kidney disease, and stroke—that collectively increase healthcare utilization and costs [5,6,7,8,9]. These complications not only elevate mortality rates but also often necessitate prolonged length of hospital stays (LOHS), adding stress to healthcare systems and diminishing patients’ quality of life.

A variety of factors have been proposed to influence the hospital trajectory of hypertensive patients. Existing research underscores the importance of concomitant conditions such as diabetes mellitus, dyslipidemia, or obesity, each of which can considerably complicate a patient’s clinical course [10,11,12]. Indeed, the interplay of metabolic disorders can weaken cardiovascular function and impede therapeutic interventions, leading to extended inpatient care [13]. Notably, nutritional status and body mass index (BMI) have garnered increased attention as potentially modifiable determinants of both hypertension severity and the associated odds of LOHS [14,15,16,17]. On the one hand, undernutrition may impair immune competency and hinder recovery, thereby prolonging hospitalization; on the other hand, overweight and obesity are often associated with chronic inflammation, endothelial dysfunction, and various metabolic derangements, all of which can exacerbate high blood pressure and prolong the LOHS or increase risk of mortality [18,19,20,21].

Although numerous studies have examined how obesity or specific comorbidities affect cardiovascular outcomes, less is known about the complex, multifactorial interactions among these variables in hypertensive populations. Emerging data suggest that sex-based differences in HT presentation and management may also play a role in hospital outcomes, further complicating efforts to develop standardized treatment algorithms [22,23,24,25]. Comprehensive multivariate analyses that account for these overlapping factors are crucial to identifying subgroups at particularly high risk for extended hospitalization. Such an approach not only refines prognostic models but can also inform tailored interventions to optimize the course of inpatient treatment [26,27].

The aim of this study was to evaluate the prognostic impact of selected factors on the risk of prolonged LOHS among patients admitted to a cardiology department with a primary diagnosis of arterial hypertension. Special emphasis was placed on applying multivariate statistical methods to identify interactions between variables and improve early patient risk assessment.

## 2. Materials and Methods

This retrospective study examined the medical records of 356 patients who were exclusively admitted as emergency cases due to arterial hypertension (ICD-10: I10) in the Cardiology Department of the University Hospital in Wroclaw (Poland) from January 2017 to June 2021. The investigation was carried out in accordance with the Strengthening the Reporting of Observational Studies in Epidemiology (STROBE) guidelines [28].

### 2.1. Study Population

All individuals admitted with a primary diagnosis of arterial hypertension, aged 18 or older, with complete data on key clinical and laboratory parameters were included in the analysis. Out of the initial 583 patient records reviewed, 227 cases were excluded due to missing data in at least one of the following variables: hypertension severity, body mass index (BMI), triglycerides (TG), low-density lipoprotein cholesterol (LDL-c), high-density lipoprotein cholesterol (HDL-c), total cholesterol (TC), high-sensitivity C-reactive protein (hsCRP), thyroid-stimulating hormone (TSH), serum potassium (K), and sodium (Na). Consequently, 356 patients met the inclusion criteria. The collected information encompassed sex, age, hypertension severity (classified according to the European Society of Cardiology) [29,30], and the presence of comorbidities such as heart failure (HF, ICD10:I50), type 2 diabetes mellitus (DM2), chronic kidney disease (CKD), cerebral stroke (CS), and myocardial infarction (MI). Laboratory parameters included TG, LDL-c, HDL-c, TC, hsCRP, TSH, K, Na, and glycosylated hemoglobin (HbA1c). The length of hospital stay (in days) was also recorded. BMI was assessed upon admission by the attending physician, and patients were categorized according to the World Health Organization’s thresholds: Underweight (<18.5 kg/m^2^), Normal weight (18.5–24.9 kg/m^2^), Overweight (25–29.9 kg/m^2^), and Obese (≥30 kg/m^2^). Data on comorbidities and BMI were collected during the initial patient evaluation, based on medical records. Laboratory parameters were obtained from the first blood samples taken upon admission. All data on comorbidities and BMI were collected by medical personnel during the admission process, based on initial patient evaluation and medical records. Laboratory parameters were obtained from the first blood samples taken upon admission. Socio-economic status and other patient-reported characteristics were not available in the dataset, as this study was based exclusively on clinical and laboratory data documented at hospital admission.

### 2.2. Data Analysis

Data preprocessing and statistical analysis were performed in Statistica 13.3 on the license of Wroclaw Medical University. Statistical inference was based on α = 0.05. Qualitative variables (features) were described with counts and frequencies, whereas median, first quartile, and third quartile were used for quantitative variables. The Mann-Whitney U test was used for between-group comparisons in values of quantitative variables. Differences in qualitative variables were handled with the use of the χ2 test. Logistic regression was used to analyze the potential multivariate association between the chosen features and the odds of prolonged in-hospital stay, defined as when hospitalization is longer than 5 days (3rd quartile in the population sample). Quantitative features were centered with values close to the median value from the population sample to improve the fit of the models by increasing the relevance of intercepts in the data context. Qualitative features were dummy-coded (producing n − 1 features, where n is the count of categories of the original a feature). Linearity vs. log(odds) was checked using the Box-Tidwell test and graphically. Although this test indicated a violation of this assumption by LDL-c (*p* = 0.025), the decision to use this variable in multivariate modeling was made based on the fact that the violation of the test was associated with unusual log(odds) at very low LDL-c values (approximately 35–60 mg/dL, Figure S1) of very slim chances to be observed in the general population, unless cholesterol-lowering therapy was involved. Possible multicollinearity cases between quantitative feature values were identified by checking the Spearman correlation (Table S1). Since an increase in total cholesterol was associated with the increase of LDL-c (ρ = 0.695), total cholesterol was excluded from multivariate analysis. Key features were selected through stepwise bi-directional elimination, in which the features were excluded from the model based on the Wald statistic and re-included based on the Lagrange multiplier (Score) test (Table S2). The resulting multivariate model (Table S3, model 1) was used in the analysis of interactions, in which a statistically significant interaction was identified through stepwise elimination from the model based on the likelihood ratio 3 test, starting from the model with all the possible interactions included (alongside with the features interacting with each other). This analysis yielded one interaction which was included in the model (Table S3, model 2). Based on the Akaike Information Criterion (AIC), deviance (D), and learning/testing AUC from 10-fold cross-validation performed on the whole dataset, the model with the interaction present performed slightly better compared to its predecessor. Hence, it was further described in the ‘Results’ section.

## 3. Results

### 3.1. Study Population

A total of 356 patients with arterial hypertension were included in the analysis. The median age was 64 years (IQR: 55–71), and the median BMI was 28.7 kg/m^2^ (IQR: 25.0–32.3). The group included 54.8% women (n = 195) and 45.2% men (n = 161). The most common hypertension grade was stage 2 (60.96%), and the mean hospitalization length was 2 days (IQR: 1–5). Detailed characteristics of the study population are shown in Table 1.

### 3.2. Univariate Differences in Context of Prolonged Hospitalization Length

Individuals of prolonged stay showed lower values of LDL-c (*p* < 0.001) and HDL-c (*p* = 0.038) and higher hsCRP (*p* = 0.024) compared to individuals of normal-length hospitalization. Although the observed frequency of the highest HT level was notably higher in the prolonged stay group, the difference was on the brink of statistical significance (*p* = 0.057). Moreover, prolonged stay was associated with a higher frequency of HF (*p* = 0.041) and MI (*p* = 0.025). Descriptive statistics are shown in Table 2 and Table 3.

### 3.3. Multivariate Analysis of the Association Between the Odds of Prolonged In-Hospital Stay and the Analyzed Variables (Features)

**Figure 1:**
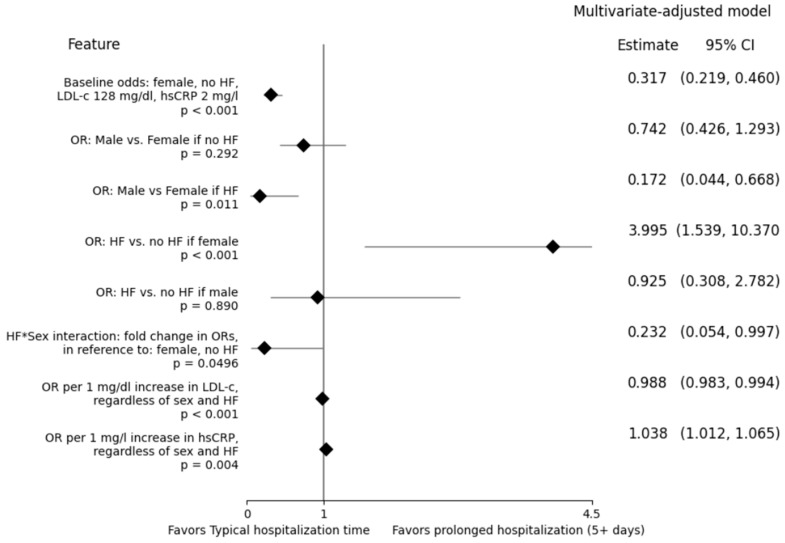
Forest plot featuring baseline odds, odds ratios (OR), and a fold odds ratio associated with prolonged hospitalization, according to the selected multivariate model (Table 4).

The multivariate model (Table 4, Table S3 model 2) utilized information on sex, HF, LDL-c, and hsCRP, with an additional correction for interaction between sex and HF. The baseline odds for a female individual with no HF and ‘typical’ LDL-c and hsCRP levels (128 mg/dL and 2 mg/L, respectively) were 0.317 (*p* < 0.001), indicating a 24.07% probability of prolonged in-hospital stay. Regardless of the sex and HF of said individual, the odds increased by 3.80% with every 1 mg/L increase in hsCRP (*p* = 0.004) and decreased by 1.21% with every 1 mg/dL increase in LDL-c (*p* < 0.001). Statistical significance of HF and sex depended on the context due to a statistically significant interaction between the two (*p* = 0.0496). Although the difference between the two sexes was statistically insignificant among non-HF individuals (*p* = 0.292), men showed approximately 5.81-fold lower odds compared to women if they both suffered from HF (*p* = 0.011). Moreover the odds were 3.995-fold higher between HF and non-HF female individuals (*p* < 0.001) but statistically insignificant if both compared individuals were male (*p*= 0.890). Odds ratios (ORs) and their associated 95% confidence intervals are shown in Figure 1.

## 4. Discussion

In this retrospective study, we showed that decreased LDL-C, increased hsCRP, and heart failure (especially in women) were predictors of prolonged LOHS in patients with hypertension.

LOHS directly affects the burden on the state budget and the health care system (the longer the hospitalization, the longer the queues for admission to the department). Hence, simple biomarkers are sought to help identify patients whose hospitalization will be longer. In our study, we identified simple biomarkers—LDL-C and hsCRP, which are routinely measured in most patients admitted to cardiology departments. Lipid disorders, mainly elevated LDL-C levels, are the most common cardiovascular risk factor in Poland (approximately 18 million Poles) [31]. Currently used lipid-lowering treatment allows for the reduction of LDL-C to very low levels, which is desirable [31]. This is due to the fact that the relationship between the risk of atherosclerotic cardiovascular disease (ASCVD) and LDL-C levels is linear, and there is no threshold below which the patient would not derive further clinical benefits (there is no plateau effect in this relationship) [31]. Hence, the current guidelines for lipid-lowering treatment emphasize the “the lower, the better” principle, which means that the lower the patient’s LDL-C level, the more beneficial it is [31]. In our study, patients with lower LDL-C levels were characterized by a longer hospitalization time. This is a form of the “cholesterol paradox”, where lower cholesterol levels are associated with worse outcomes [32]. Most likely, these were patients treated for hypercholesterolemia, in whom other diseases, including those of atherosclerotic etiology, co-occurred. Other studies have shown that an extended analysis of the clinical condition of patients caused the “cholesterol paradox” to cease to exist [33]. In general, the LOHS of patients with chronic diseases is longer, which has been shown in other studies [34]. In older adults, lower cholesterol levels may be indicative of poorer baseline health and nutritional status, which may be another explanation for the observed relationship (lower LDL-C level—longer hospitalization) [32]. Malnutrition, which is relatively often found in older patients with hypertension, eliminates the observed “cholesterol paradox” [35,36]. Low LDL-C levels may be the effect of effective lipid-lowering treatment on the one hand, and on the other hand, it may be a biomarker of malnutrition in adults and the elderly [37]. Researchers studying nutritional status confirm that malnutrition upon hospital admission is quite common and is associated not only with LOHS but also with a worse prognosis [38]. Other factors unrelated to the functioning of the cardiovascular system may also play an important role in modulating these relationships [36]. Another biomarker that was associated with longer hospitalization in our study was elevated hsCRP levels. Elevated CRP levels are associated with a poorer prognosis and a higher risk of cardiovascular and all-cause death [39]. Elevated hsCRP levels indicate the presence of low-grade inflammation, which contributes to the occurrence of many diseases, not only cardiovascular diseases, and may also affect the general condition of hospitalized patients with arterial hypertension [40]. Hence, patients with hypertension and increased CRP levels may be more burdened with multi-morbidities. Moreover, increased CRP levels may be a biomarker of insufficient control of various diseases, e.g., diabetes [41]. It is worth noting that CRP level is significantly influenced by eating habits and the nutritional status of the body [42]. In patients with malnutrition, increased CRP level is observed [43]. In our study, low LDL-C and increased CRP levels, i.e., biomarkers of poor nutritional status (undernutrition), were associated with longer hospitalization time. Our observations have significant clinical significance because they indicate which patients should be assessed more thoroughly in terms of nutritional status. It is worth remembering that BMI measurement and body weight, in general, do not reflect the actual nutritional status [44,45]. Over 50% of patients with obesity have nutritional deficiencies [44]. In our study, we did not demonstrate the effect of BMI on the LOHS.

An important factor that prolonged LOHS, especially in women, was HF (3.995-fold higher odds of prolonged LOHS). HF is an advanced stage of the cardiovascular continuum and is generally associated with a worse prognosis [46]. In women and men, hypertension increases the odds of HF by 3- and 2-fold, respectively. Hypertensive heart disease is responsible for roughly one-fourth of all causes of HF [46]. Although hypertension remains more common in males, the gradient by which hypertension develops across the lifespan in females is steeper, while the blood pressure thresholds at which cardiovascular diseases develop are lower. These differences may be related to sex-specific risk factors such as hypertensive disorders of pregnancy and menopause [24]. An important factor influencing the differences in the length of hospitalization may also result from the more frequent occurrence of metabolic syndrome in women, differences in the pharmacokinetics and pharmacodynamics of drugs, and changes in the hormonal system. All of this may significantly influence the observation that women with HF have a longer hospitalization time compared to men with HF [47,48]. It is also worth emphasizing that despite the fact that cardiovascular disease kills more women than any other cause, women may receive less aggressive treatment for risk factors and are often not treated with the recommended guidelines for a myriad of cardiovascular disease diagnoses [49]. Nevertheless, the results of our study indicate that the presence of heart failure in women with hypertension is a predictor of longer hospitalization.

The number of studies that have attempted to identify predictors of LOHS in cardiology departments is not large. In the study by García-González et al., the risk factors for LOHS (>4 days of hospitalization) in the cardiology department included age, Friday admission, heart failure, and creatinine concentration [50]. The LOHS risk factors identified in our and other studies can be used to develop a simple model based on artificial intelligence that will help identify patients at increased risk of LOHS. In an interesting study by Daghistani et al., based on simple parameters such as admission heart rate, on-admission systolic and diastolic blood pressure, age, and insurance status (eligibility), a fairly accurate prediction of LOHS was achieved using machine learning (sensitivity 0.80, accuracy 0.80, and AUROC 0.94) [51]. This approach allows for the identification of patients who, in order to optimize the time of hospitalization, may require special care from medical personnel [52]. Similar algorithms were used with good results not only among patients admitted to cardiology departments but also to the emergency department [53]. Our study complements the knowledge of LOHS predictors in hospitalized patients with hypertension with LDL-C and hsCRP, which may additionally increase the precision of such algorithms in the future.

In the context of patients with concomitant heart failure, observations from the study by Ignatavičiūtė et al. can be used, in which parameters such as treatment interruption, higher value of NT-proBNP, estimated glomerular filtration rate (eGFR) ≤ 50 mL/min/1.73 m^2^, systolic blood pressure (BP) ≤ 135 mmHg, and severe tricuspid valve regurgitation were additional predictors of LOHS [54]. This may allow for an even more accurate prediction of LOHS in this special group of patients.

It should also be emphasized that some of the above-mentioned LOHS risk factors are also risk factors for in-hospital mortality (age, sex, diagnosis, comorbidities, mode of admission (urgent versus elective), need for transfer between hospitals, number of previous emergency admissions, and LOHS were the most relevant patient-related factors for in-hospital mortality [55]).

It is worth noting that patients admitted to cardiology departments, in most cases, undergo tests that were predictors of LOHS in our and other studies. Therefore, analysis of these parameters does not generate additional costs in most cases and may even contribute to better management of patients at risk of LOHS and, consequently, optimize the costs of hospitalization of such patients. A good example is in hospital infections. It is known that LOHS is significantly associated with a higher risk of in-hospital infection, and their occurrence additionally prolongs the duration of hospitalization and worsens the prognosis of patients [56]. Therefore, the identification of patients at particularly high risk of LOHS at the earliest possible stage of hospitalization may allow for the implementation of appropriate procedures to optimize the risk of in-hospital infection.

### 4.1. Study Limitation

This study has some limitations that should be considered when interpreting the results. Due to its retrospective design, detailed information regarding the use of cholesterol-lowering medications or other therapies potentially influencing LDL-c and hsCRP levels was not available. Similarly, the lack of data on specific antihypertensive regimens and other treatments administered during hospitalization could have influenced the observed outcomes. Furthermore, as a single-center study, the findings may not fully reflect the diversity of patients with HT across different healthcare settings or populations.

The sample size, while sufficient for the statistical analysis performed, may not account for the full variability of clinical presentations in patients with HT and related comorbidities, such as HF. In addition, HF status was recorded only as a binary variable (present/absent) at hospital admission. In addition, HF status was recorded only as a binary variable (present/absent) at hospital admission, with no differentiation into specific heart failure types. Consequently, we were unable to assess the prognostic impact of EF on prolonged LOHS. Similarly, NT-proBNP levels were not routinely measured in patients with hypertension at hospital admission, and therefore, we could not include this parameter in our analysis. Although significant interactions, such as the relationship between sex and HF, were identified, it is possible that other clinically relevant interactions were not explored. Additionally, the observational nature of the study does not allow for causal inferences regarding the relationships between LDL-c, hsCRP, and LOHS.

Future prospective studies are recommended to validate these findings in larger, more diverse populations. Such studies should include detailed information on medication use, treatment strategies, and additional clinical factors to provide a more comprehensive understanding of the predictors of prolonged LOHS in patients with HT. These efforts will help refine risk stratification and guide personalized management strategies in this patient population.

### 4.2. Implications for Nursing Practice

The findings of this study emphasize the need for a proactive and individualized approach in nursing care for patients with HT. Nurses should prioritize the early identification of patients at risk of prolonged length of hospital stay (LOHS). Given their direct role in patient monitoring and care coordination, nurses are in a unique position to recognize clinical and socioeconomic factors that may contribute to extended hospitalization. Implementing early interventions, such as structured discharge planning and patient education, can help optimize hospital resource utilization and improve patient outcomes.

A key implication for nursing practice is the development of patient-centered care strategies that account for individual risk factors, such as comorbidities and demographic differences. For example, nursing care plans for patients with HF should address potential complications and include enhanced monitoring protocols for high-risk groups, such as female patients. Furthermore, nurses can play a critical role in managing heart failure patients through targeted interventions, including early initiation of diuretic therapy, close monitoring of NT-proBNP levels, and stratification of HF subtypes to guide treatment adjustments.

Additionally, nurses play a fundamental role in patient education and advocacy. Structured education programs focusing on hypertension self-management, lifestyle modifications, and medication adherence can significantly impact hospital readmissions and overall disease progression. Evidence suggests that multidisciplinary, nurse-led interventions can improve blood pressure control, reduce cardiovascular complications, and, ultimately, shorten hospital stays [57,58]. Empowering patients with knowledge and resources can help reduce the odds of complications and improve long-term outcomes.

This study also underscores the importance of interdisciplinary collaboration in patient care. Nurses should actively engage in care planning and communicate findings related to patients’ laboratory and clinical profiles to the broader healthcare team. This collaborative approach ensures that care strategies are aligned and comprehensive. Specifically, by addressing factors such as nutritional status and inflammatory markers (LDL-C, hsCRP), nurses can contribute to the early identification of high-risk patients and assist in clinical decision-making to prevent unnecessary hospitalization.

Finally, nurses should advocate for evidence-based practice standards that reflect the complex needs of hypertensive patients in acute care settings. Given their frontline role in inpatient care, nurses should also be involved in quality improvement initiatives aimed at reducing prolonged hospitalizations, such as implementing weekend discharge protocols and addressing chronobiological patterns of discharge delays. This study highlights the significant role of nurses in ensuring efficient, patient-centered care for hypertensive individuals in cardiology departments.

## 5. Conclusions

This study identified significant predictors of prolonged LOHS in patients admitted with HT. Lower LDL-c levels and elevated hsCRP concentrations were associated with increased odds of extended LOHS. Additionally, a significant interaction between sex and HF was observed. Specifically, female patients with HF demonstrated nearly four-fold higher odds of prolonged LOHS compared to females without HF, while this relationship was not observed in male patients. These findings highlight the importance of considering both biochemical markers and sex-specific interactions in assessing odds and optimizing management strategies for patients with HT. Future prospective research involving more diverse patient populations is needed to validate and expand these observations, ultimately informing more personalized approaches to the management of hypertensive patients.

## Figures and Tables

**Table 1:** Overall characteristics of the population sample.

Quantitative Variables						
Variable	n	Me	1Q	3Q	Min	Max
Age [years]	356	64.00	55.00	71.00	22.00	93.00
BMI	356	28.71	25.03	32.33	14.42	48.07
TG [mg/dL]	356	117.00	88.00	153.50	37.00	433.00
LDL-c [mg/dL]	356	133.00	89.00	169.00	35.00	370.00
HDL-c [mg/dL]	356	52.00	44.00	61.50	9.00	106.00
TC [mg/dL]	356	183.00	153.00	218.50	77.00	415.00
hsCRP [mg/L]	356	1.97	0.97	3.88	0.16	321.25
TSH [µIU/mL]	356	1.34	0.88	2.06	0.01	8.73
K [mmol/L]	356	4.22	3.96	4.49	3.06	7.02
Na [mmol/L]	356	140.00	139.00	142.00	127.00	152.00
HbA_1C_ [%]	258	5.80	5.50	6.30	4.30	10.70
Hospitalization length [days]	356	3.00	1.00	5.00	1.00	21.00
Qualitative variables						
Variable: category	n	%				
Sex: female	195	54.78				
Sex: male	161	45.22				
HT level: 1	73	20.51				
HT level: 2	217	60.96				
HT level: 3	66	18.54				
HF: Yes	46	12.92				
DM2: Yes	101	28.37				
CKD: Yes	48	13.48				
CS: Yes	46	12.92				
MI: Yes	21	5.90				

Abbreviations: BMI, body mass index; CKD, chronic kidney disease; CS, cerebral stroke; DM, diabetes mellitus; HF, heart failure; HDL-c, high-density lipoprotein cholesterol; HbA1c, hemoglobin A1c; hsCRP, high-sensitivity C-reactive protein; HT, hypertension; K, potassium; LDL-c, low-density lipoprotein cholesterol; LOHS, length of hospital stay; MI, myocardial infarction; Na, sodium; TC, total cholesterol; TG, triglycerides; TSH, thyroid-stimulating hormone.

**Table 2:** Characteristics of the population sample upon stratification by the fact of prolonged hospital stay—Quantitative variables.

Variable	Normal Hospital Stay Length						Prolonged (≥5 Days) Hospital Stay						*p*
	n	Me	1Q	3Q	Min	Max	n	Me	1Q	3Q	Min	Max	
Age [years]	261	64.000	55.000	70.000	22.00	91.00	95	66.000	56.000	73.000	26.00	93.00	0.334
BMI	261	28.730	25.150	32.180	18.83	48.07	95	28.260	24.220	32.460	14.42	41.97	0.835
TG [mg/dL]	261	117.000	86.000	153.000	43.00	433.00	95	118.000	92.000	154.000	37.00	390.00	0.825
LDL-c [mg/dL]	261	139.000	94.000	175.000	35.00	370.00	95	109.000	79.000	139.000	43.00	233.00	<0.001
HDL-c [mg/dL]	261	53.000	45.000	62.000	27.00	106.00	95	50.000	41.000	58.000	9.00	89.00	0.038
TC [mg/dL]	261	184.000	153.000	220.000	84.00	415.00	95	183.000	152.000	218.000	77.00	329.00	0.674
hsCRP [mg/L]	261	1.840	0.910	3.440	0.16	55.24	95	2.640	1.130	6.010	0.17	321.25	0.024
TSH [µIU/mL]	261	1.340	0.940	2.060	0.08	8.73	95	1.410	0.800	2.130	0.01	7.66	0.877
K [mmol/L]	261	4.230	3.970	4.480	3.12	7.02	95	4.190	3.930	4.560	3.06	6.08	0.844
Na [mmol/L]	261	140.000	139.000	142.000	130.00	148.00	95	140.000	138.000	141.000	127.00	152.00	0.118
HbA_1C_ [%]	168	5.800	5.500	6.200	4.30	10.00	90	5.850	5.400	6.300	4.70	10.70	0.871
Hospitalization length [days]	261	2.000	1.000	3.000	1.00	4.00	95	6.000	5.000	8.000	5.00	21.00	-

Abbreviations: BMI, body mass index; TG, triglycerides; LDL-c, low-density lipoprotein cholesterol; HDL-c, high-density lipoprotein cholesterol; TC, total cholesterol; hsCRP, high-sensitivity C-reactive protein; TSH, thyroid-stimulating hormone; K, potassium; Na, sodium; HbA1C, hemoglobin A1c.

**Table 3:** Characteristics of the population sample upon stratification by the fact of prolonged hospital stay—Qualitative variables.

Variable	Normal Hospital Stay Length		Prolonged (≥5 Days) Hospital Stay		*p*
	N	Frequency	n	Frequency	
Sex (female)	137	0.525	58	0.611	0.151
Sex (male)	124	0.475	37	0.389	
HT level: 1	53	0.203	20	0.211	0.057
HT level: 2	167	0.640	50	0.526	
HT level: 3	41	0.157	25	0.263	
HF: No	233	0.893	77	0.811	0.041
HF: Yes	28	0.107	18	0.189	
DM2: No	190	0.728	65	0.684	0.418
DM2: Yes	71	0.272	30	0.316	
CKD: No	229	0.877	79	0.832	0.263
CKD: Yes	32	0.123	16	0.168	
CS: No	229	0.877	81	0.853	0.538
CS: Yes	32	0.123	14	0.147	
MI: No	250	0.958	85	0.895	0.025
MI: Yes	11	0.042	10	0.105	

Abbreviations: HT, hypertension; HF, heart failure; DM, diabetes mellitus; CKD, chronic kidney disease; CS, cerebral stroke; MI, myocardial infarction.

**Table 4:** Key information from the chosen multivariate logistic regression model—association between selected features and the odds of prolonged in-hospital stay.

Estimates Directly Based on the Model					
Feature	Interpretation	*p*-Value	Estimate	Estimate −95% CI	Estimate 95% CI
A. Intercept	The odds for a female individual, with no HF, LDL-c 128 mg/dL, and hsCRP 2 mg/L	<0.001	0.317	0.219	0.460
B: Sex: Male	The fold change in (A) [OR] if the individual was male	0.292	0.742	0.426	1.293
C: HF: Yes	The fold change in (A) [OR] if the individual suffered from HF	<0.001	3.995	1.539	10.370
LDL-c c 128	The fold change in (A) [OR] upon each 1 mg/dL increase in LDL-c	<0.001	0.988	0.983	0.994
hsCRP c 2	The fold change in (A) [OR] upon each 1 mg/L increase in hsCRP	0.004	1.038	1.012	1.065
Sex*HF	The fold difference in (B) between individuals with HF and those without HF… or… The fold difference in (C) between male and female individuals	0.0496	0.232	0.054	0.997
Additional estimates, associated with the Sex*HF interaction					
Feature	Interpretation	*p*-Value	Estimate	Estimate −95% CI	Estimate 95% CI
Sex: Male|HF = Yes	The fold difference in baseline odds between male and female individuals with HF	0.011	0.172	0.044	0.668
HF: Yes|Sex = Male	The fold difference in baseline odds between male HF and non-HF individuals	0.890	0.925	0.308	2.782

The ‘c: x’ labels in case of continuous features indicate centering of the feature at value x (so that x becomes 0) in order to increase the interpretability of the ‘Intercept’ feature. Abbreviations: CI, confidence interval; CRP, C-reactive protein; HF, heart failure; LDL-c, low-density lipoprotein cholesterol; OR, odds ratio.

## Data Availability

The data can be accessed by contacting the corresponding author.

## Supplementary Materials

The following supporting information can be downloaded at: https://www.mdpi.com/article/10.3390/nursrep15030110/s1, Figure S1. Exploring the assumption of linearity vs. log(odds) in case of LDL-c; Table S1. Spearman correlation coefficient (ρ) matrix—identifying possible multicollinearity between the predictors; Table S2. The stepwise process of multivariate key feature selection through bi-directional elimination; Table S3. A more thorough information on the multivariate logistic regression models analyzed in this study.

## Guidelines and Standards Statement

This study followed the STROBE (Strengthening the Reporting of Observational Studies in Epidemiology) guidelines to ensure the reliability and clarity of the observational study reporting.

## Abbreviations

The following abbreviations are used in this manuscript:

BMI	Body Mass Index
CKD	Chronic Kidney Disease
CS	Cerebral Stroke
DM2	Type 2 Diabetes Mellitus
HDL-c	High-Density Lipoprotein Cholesterol
HbA1c	Hemoglobin A1c
hsCRP	High-Sensitivity C-Reactive Protein
HT	Hypertension
K	Potassium
LDL-c	Low-Density Lipoprotein Cholesterol
LOHS	Length of Hospital Stay
MI	Myocardial Infarction
Na	Sodium
TC	Total Cholesterol
TG	Triglycerides
TSH	Thyroid-Stimulating Hormone
